# Machine Learning Methods Applied to Predict Ventilator-Associated Pneumonia with *Pseudomonas aeruginosa* Infection via Sensor Array of Electronic Nose in Intensive Care Unit

**DOI:** 10.3390/s19081866

**Published:** 2019-04-18

**Authors:** Yu-Hsuan Liao, Zhong-Chuang Wang, Fu-Gui Zhang, Maysam F. Abbod, Chung-Hung Shih, Jiann-Shing Shieh

**Affiliations:** 1 Department of Mechanical Engineering, Yuan Ze University, Chungli 32003, Taiwan; comimic@gmail.com (Y.-H.L.); wangzhchuang@126.com (Z.-C.W.); 2 College of Mechanical Engineering, Guizhou University, Guiyang 550025, China; zhfugui@vip.163.com; 3 College of Engineering, Design and Physical Sciences, Brunel University London, Uxbridge UB8 3PH, UK; Maysam.Abbod@brunel.ac.uk; 4 Division of Thoracic Medicine, Department of Internal Medicine, School of Medicine, College of Medicine, Taipei Medical University, Taipei 11031, Taiwan; 5 Division of Pulmonary Medicine, Department of Internal Medicine, Taipei Medical University Hospital, Taipei 11031, Taiwan

**Keywords:** intensive care unit, ventilator-associated pneumonia, artificial neural network, support vector machine, ensemble neural networks, cross-validation

## Abstract

One concern to the patients is the off-line detection of pneumonia infection status after using the ventilator in the intensive care unit. Hence, machine learning methods for ventilator-associated pneumonia (VAP) rapid diagnose are proposed. A popular device, Cyranose 320 e-nose, is usually used in research on lung disease, which is a highly integrated system and sensor comprising 32 array using polymer and carbon black materials. In this study, a total of 24 subjects were involved, including 12 subjects who are infected with pneumonia, and the rest are non-infected. Three layers of back propagation artificial neural network and support vector machine (SVM) methods were applied to patients’ data to predict whether they are infected with VAP with *Pseudomonas aeruginosa* infection. Furthermore, in order to improve the accuracy and the generalization of the prediction models, the ensemble neural networks (ENN) method was applied. In this study, ENN and SVM prediction models were trained and tested. In order to evaluate the models’ performance, a fivefold cross-validation method was applied. The results showed that both ENN and SVM models have high recognition rates of VAP with *Pseudomonas aeruginosa* infection, with 0.9479 ± 0.0135 and 0.8686 ± 0.0422 accuracies, 0.9714 ± 0.0131, 0.9250 ± 0.0423 sensitivities, and 0.9288 ± 0.0306, 0.8639 ± 0.0276 positive predictive values, respectively. The ENN model showed better performance compared to SVM in the recognition of VAP with *Pseudomonas aeruginosa* infection. The areas under the receiver operating characteristic curve of the two models were 0.9842 ± 0.0058 and 0.9410 ± 0.0301, respectively, showing that both models are very stable and accurate classifiers. This study aims to assist the physician in providing a scientific and effective reference for performing early detection in *Pseudomonas aeruginosa* infection or other diseases.

## 1. Introduction

In Taiwan, hundreds of people are admitted to the intensive care unit (ICU) for various reasons every day. However, the lower respiratory tract of patients is very susceptible to be infected and even cause pneumonia after using the ventilators in the ICU. Ventilator-associated pneumonia (VAP) [1,2,3] has a mortality rate of 20–50%, and in some areas even higher. VAP is one of the top ten hospital infections, and is the third largest causes of death [4]. Traditional VAP diagnoses methods, including chest X-ray, may not be reliable unless the symptoms are severe. Sputum culture can be an easier way to detect VAP, but growing the bacterial culture in general is a time consuming process, which requires at least 5–7 days. Sometimes, patients’ conditions can deteriorate during this crucial time, leading to death. In recent years, the application of respiratory analysis has received increasing attention in medicine, which not only serves as a diagnostic tool, but also to monitor the progress of therapies [5,6]. In addition, the e-nose represents an easy and low cost method for exhaled volatile compound analysis, and distinguishes pulmonary disorders from health [7]. In fact, many kinds of diseases such as renal disease [8,9], skin disease [10,11], diabetes [12,13], lung cancer [14,15], asthma [16], and obstructive sleep apnea (OSA) [17] can be diagnosed by electronic nose, as illustrated in Table 1.

Recently, breath gases have been applied to predict pneumonia infections [18,19]. Paramedics have noted that patients infected with VAP have significant odors, which provides a possibility for rapid detection of pneumonia. Pneumonia can be identified more easily by detecting odors [20]. Analysis of Volatile Organic Compounds (VOCs) in exhaled breath, and thermal desorption with gas chromatography coupled to mass spectrometry (TD-GC-MS) can be used to identify VOCs and the causative pathogen in patients suspected of VAP [21,22,23]. However, researchers have found that a variety of bacteria such as *Klebsiella pneumoniae, Acinetobacter baumanii, Escherichia coli, Staphylococcus aureus*, and *Pseudomonas aeruginosa* contribute to VAP, and it is very crucial to find a way to recognize these different types. Therefore, Schnabel et al. have identified the promising role of electronic nose in predicting whether patients are infected with VAP [24]. On the other hand, Wong et al. [25] developed a rapid VAP detection system, which features a nose-on-chip that detects VAP with a very high accuracy rate. Using data from 143 pneumonia patients, and k-nearest neighbor classifier (KNN) has achieved an identification rate of pneumonia about 75%. Scott et al. [26] systematically introduced data analysis methods using electronic nose system, including data normalization methods, pattern recognition and classification algorithms. In the past, the artificial neural network (ANN) [27,28] has been proposed as an appropriate algorithm suitable for modeling complex nonlinear relationships in medical research. ANN can also be operated in real time, supplying strong tolerance of faults. Recently, in order to generate a general model, ensemble neural networks (ENN) is used by combining different models from the same data set [29]. Support vector machine (SVM) is another machine learning method proposed according to statistical theory [30], which is applied for the feature classification and regression. SVM shows many unique advantages in solving a small number of samples, non-linear, and high-dimensional pattern recognition problems, and is also easy to use [31,32,33]. Therefore, ENN and SVM models are the best known and most evolved classification algorithms for electronic noses. Finally, the main goal of this paper is to collect patients’ breath gases data and build ENN and SVM prediction models to predict whether patients are infected with VAP. It can also be examined via receiver operating characteristic curves (ROC). 

## 2. Materials and Methods

### 2.1. Database

There are mainly five types of pneumonia bacteria in ICUs, including *Klebsiella pneumoniae, Acinetobacter baumanii, Escherichia coli, Staphylococcus aureus*, and *Pseudomonas aeruginosa*. In this study, although several types of pneumonia bacteria were collected, the numbers of most bacteria strains were too small, in fact, only the numbers of *Pseudomonas aeruginosa* samples were large. Therefore, *Pseudomonas aeruginosa* was selected as the indicator bacteria of pneumonia.

**Figure 1:**
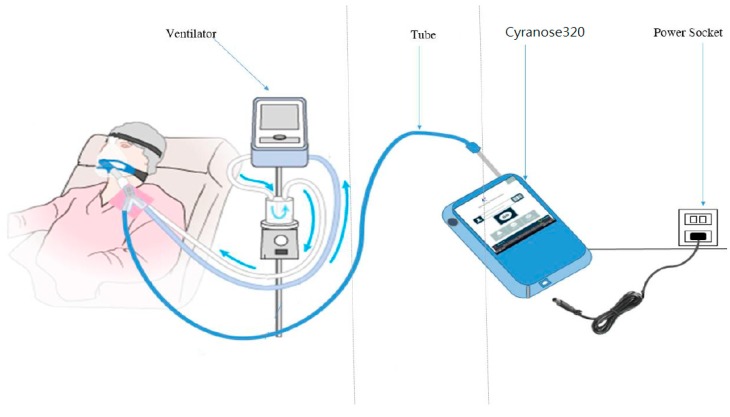
Breath gases collection in ICU (intensive care unit).

Exhaled breath is rich in many VOCs. A recent review reported 1765 volatile compounds appearing in exhaled breath. Exhaled breath contains metabolites in the gas phase called VOCs. Therefore, exhaled breath analysis can be used to identify the causative pathogen in patients suspected of VAP [34]. Schnabel et al. reported that a subset of 12 VOCs could be used to discriminate patients with VAP [35]. Cyranose-320 electronic nose (Sensigent intelligent sensing solutions) was used for patients’ breath gases data collection, which is equipped with 32 individual polymer sensors. The breath gases were collected at the end of the ventilator paths, as shown in Figure 1. The database, included a total of 24 subjects, of which, 12 subjects were the patients that admitted to the Taipei Medical University Hospital (TMUH) with pneumonia, and the others were non-infected individuals. ICU patients were mainly patients who had received neurosurgery without infection evidence and lung disease. The raw data of 140 samples were collected for each patient, totaling 3360 raw data points collected. In order to avoid bacterial variation due to the implementation of treatment, non-injected antibiotic patients’ breath gases were collected as an experimental database. To ensure patients’ safety, the sampling processes were carried out by expert medical staff, and the sampling time was limited to 10 s. The details of data collection distribution of the selected boundaries between the VAP infected and non-infected patients are shown in Table 2. The present research received the approval from the Institutional Review Board at TMUH of Taiwan. Written informed consents were obtained from the 24 patients.

### 2.2. Sensor Array Sensitivity Analysis

Fourier infrared spectrometer (FT-IR) [36] and chromatography-mass spectrometry (GC-MS) analyzer are the best and most standard methods for gas analysis [37]. Although FT-IR and GC-MS supply very high accuracy of gas identification, they are very expensive, time-consuming, and not portable, making the processes of sample analysis very complicated, requiring expert paramedics for interpretation [38]. A cheap, time saving, and portable alternative is the electronic nose. For variable gases, the electronic nose uses an array of sensors to produce different response resistance changes and acquire a specific response pattern for each type of gases, and this specific pattern is applied for disease identification [39,40]. In this paper, *Pseudomonas aeruginosa* was chosen as the indicator bacteria of pneumonia to recognize if patients were VAP infected. For each array of sensors, the larger differences of response resistances between pneumonia and non-infected pneumonia data, are easier for VAP identification. However, results logged from the same sensor do not show significant difference between pneumonia data and non-infected pneumonia data. This low recognition accuracy will have an impact on pneumonia identification models. Therefore, the sensitivity of the sensor array was analyzed first, and the sensors with low recognition accuracy were abandoned. 

**Figure 2:**
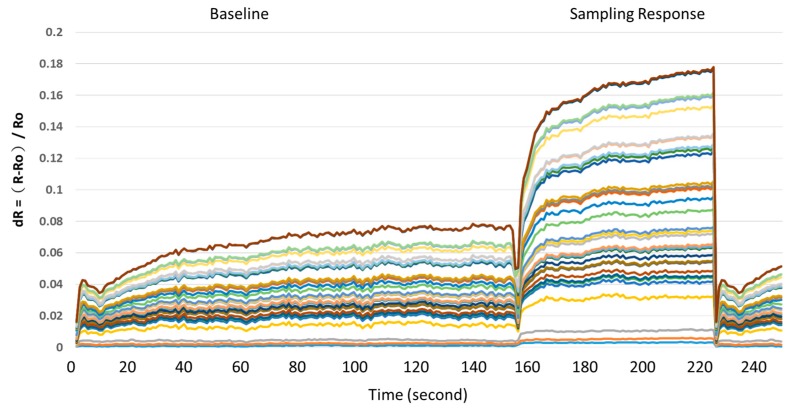
Sensors response ratio.

Figure 2 shows the collected data of a typical response of the Cyranose320 device and sensor module of 32 sensors array [41]. The first stage (around 0–150 s) was the baseline value of the room air. Then, in the second stage (around 151–230 s), when 32 sensors were exposed to breath samples, the resistance changes across the array were captured as a digital pattern that is representative of the test results. Finally, MATLAB software was used to extract features from the data saved in the Cyranose320 device using the static change in sensor resistance. In order to standardize the measurement, all data were normalized via a fractional difference model as in Equation (1), where R is the response resistance of the sensor to the *Pseudomonas aeruginosa* samples, and Ro is the baseline value of the room air using as a reference gas. Furthermore, the data set of the 32 sensors was normalized to [0~1] by dividing each dR by the maximum value for each sensor as shown in Y axis in Figure 2.

dR = (R − Ro)/Ro(1)

**Figure 3:**
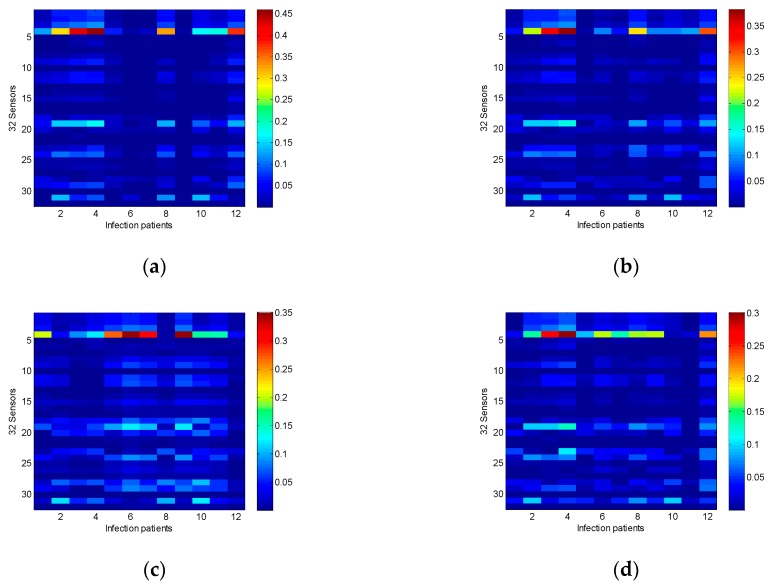

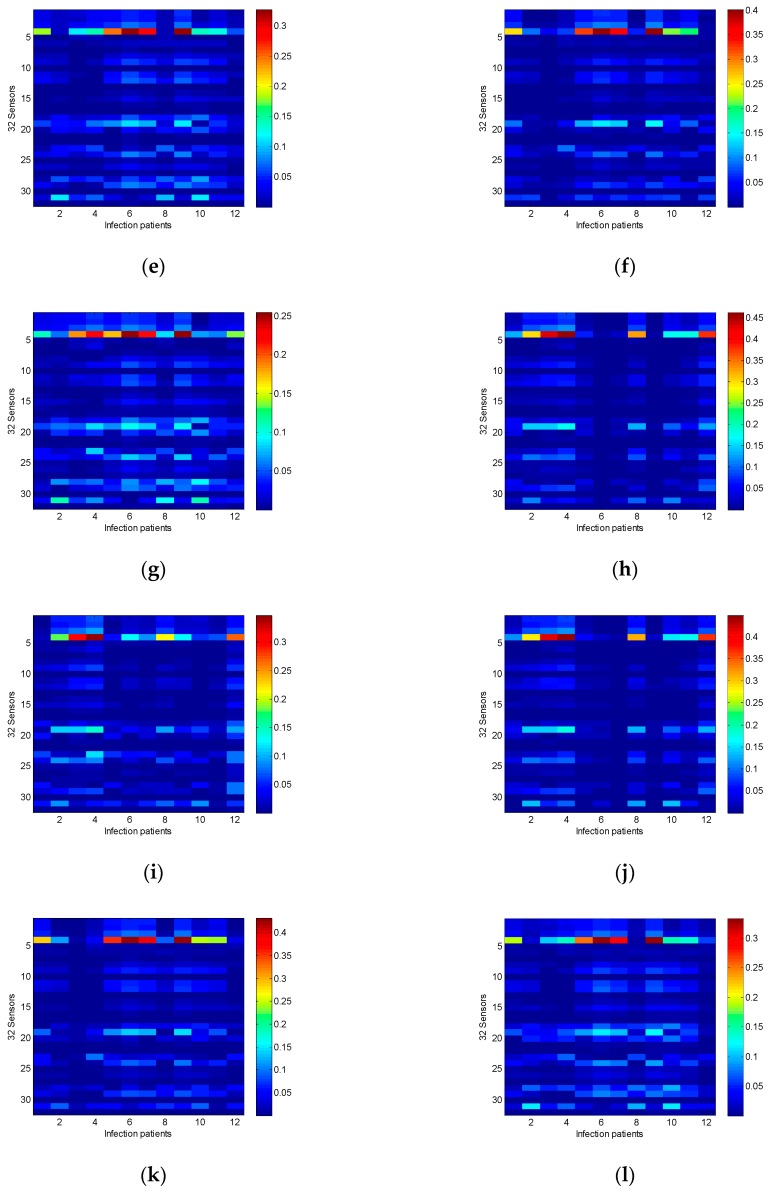
The mean absolute error of the value of sensor resistance between the data of total 12 patients infected with pneumonia and the data of each non-infected patient (i.e., from (**a**) to (**l**) of all 12 patients) in all 32 sensors.

The MATLAB command ’imagesc’ was utilized to scale image data to the full range of the current colormap and displays the image. The results are shown in form of images for comparative analysis, which are very intuitive and easy to read as shown in Figure 3. According to the colormap, the blue color presents very low and the opposite red color presents very high values. Therefore, the cost function is based on the mean absolute error (MAE) of the sensor resistance of 12 infected patients with pneumonia and non-infected patient (i.e., from (a) to (l) of Figure 3 of all 12 patients) in all 32 sensors. Results show that the differences of resistances response between pneumonia patients and non-infected patients are very small for sensors numbers 6, 10, 13, 21, 27, and 32. Therefore, this experiment excludes these six sensors since there is no significant effect on the analysis results. Removing these six sensors data from the data set can reduce the algorithm’s complexity and increase its performance. Finally, the gases data collected from the remaining 26 sensors were used to establish the pneumonia identification models.

### 2.3. Data Preprocess 

Electronic nose sensors are easily affected by noise from the environment, including temperature, humidity and other factors. Environmental factors may lead to data drift or singularity data occurrence. Besides, the range of some inputs data may be particularly large, the existence of these singular data may cause the longer training of the prediction models training, converging harder, and even reduce the accuracy of the models. For this purpose, a non-linear Log-Sigmoid activation function was used, which is defined as in Equation (2):

(2) $$a\;=\;f(n)\;=\frac{1}{1+e^{-n}}$$

where a is the output of non-linear Log–Sigmoid function and n is the input of non-linear activation function. The training and testing data are normalized in the range of 0 to 1. The following two algorithms used Equation (2) to preprocess all collected sampling data. This data preprocess method is very effective in reducing data drift for different temperature and humidity.

### 2.4. Architecture of Ensemble Neural Networks

ENN models [42,43] can be classified into many different types with different application areas, learning methods, or structures. Typically, feedforward neural network is the most established and utilized network. The network uses back-propagation for training and weights setting, and can be used for regression and classification. A typical classification network consists of an input layer, hidden layer and an output layer. The input layer size is set according to the number of features in the data, while the hidden layer is set according to the data complexity and non-linearity. Finally, the output layer is a representative of the classes, two class data sets required a single output neuron. The data are usually divided into three portions, a major portion is for training, and two small portions for validation and testing. The validation data are used during training to prevent the network from overfitting. Meanwhile, the testing data are used to check the accuracy of the fitted model to unseen data. The training process starts by initializing the network weights randomly, which sometimes might cause a problem with convergence and accuracy. Each time the neural network is trained, it can generate different model with high accuracy as specific area of the search space. In order to generate a general model, the ensemble neural network is used by combining different models from the same data set. The concept of ensemble is based on combining different models, which are trained using different initial weights to generate a generalized model.

Furthermore, the neural network generalization can be improved using the ensemble technique based on segmenting the training data. The data can be organized into segments such that cross validation is used to generate different neural network models. The concept of cross-validation is to generate k-fold cross validation of equal sizes. One data fold is used as the validation set, while the other folds are used for training to generate a neural network model. This process is repeated *k* times, where each time the validation sets is selected from the k-fold without being used twice. This process will generate *k* number of neural networks that are averaged to generate the final prediction.

**Figure 4:**
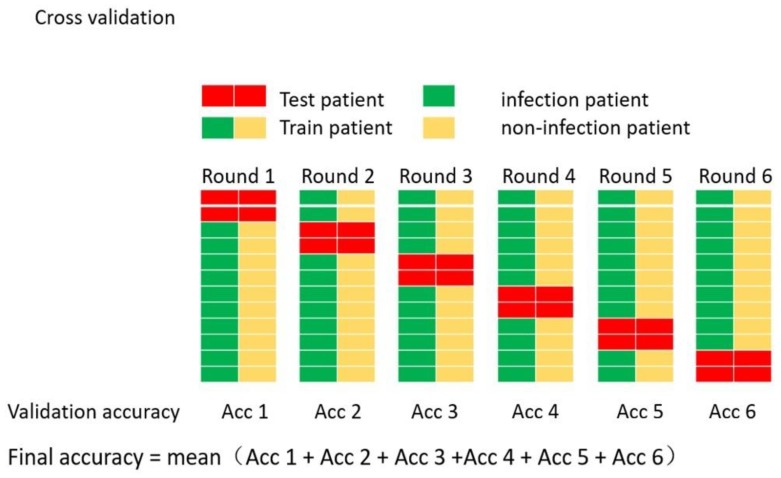
Cross-validating an ENN (ensemble neural networks) model (note: Acc means Accuracy).

The process of establishing an ensemble using the cross-validation method for creating the ENN is shown in Figure 4. The figure illustrates cross-validation where the data are divided into cyclic training and validation segments, and a fixed testing segment.

1. First, randomly select four cases from the database as the testing dataset, including two infection cases and two non-infection cases. Each case includes 140 patients’ data.
2. The rest of the database was divided into five segments (dataset1, dataset2, ..., dataset5). Each dataset includes two infection cases and two non-infection cases. Randomly select four datasets as the training datasets, the remaining dataset is the validation dataset to check the model for over-fitting. This step is repeated five times such that five different training datasets and one validation dataset are produced. Each training and validation dataset were used to train 10 networks with different initial weights.
3. After the training process is finalized, the testing dataset is applied to test the classification accuracy and generalization of the ANN classifier, then the best network in each training and validation datasets are selected to be combined into the ensemble model.
4. Finally, an ENN model constructed by five networks is established. The output of the ENN model is the average of five best networks.

### 2.5. Architecture of SVM

SVM is a discriminative classification method, which works by finding a separating hyper-plane in the feature space to distinguish data into different types. SVM models require the definition of different parameters and transfer functions to deal with different problems. In this paper, radial basis function (RBF) was selected as a kernel function [44], which is defined by Equations (3) and (4):

(3) $$K\left(x,\;x_{i}\right)=\;\exp(-\frac{{\left|x-x_{i}\right|}^{2}}{\sigma^{2}})$$

(4) $$f(x)\;=\;\mathrm{sgn}\left(\overset{l}{\underset{i=1}{\sum }}a_{i}K\left(x,x_{i}\right)+b\right)$$

RBF is the most used transfer function for SVM model. SVM contains two kinds of crucial parameters known as the kernel function parameters σ and regularization parameters C, that are very important for the performance of SVM model [45]. Reasonable parameter values can make the SVM model achieve higher training accuracy and stronger generalization ability. If C is too small or too large, the generalization ability of the SVM model may get worse. If σ is too small, the training accuracy may be high, but the testing accuracy will be pretty poor, which is a phenomenon known as over-fitting. However, when using too large σ, the SVM model cannot get particularly high accuracy either. Above all, for the SVM model to have the highest classification performance, the most suitable C and σ parameters must be selected first.

**Figure 5:**
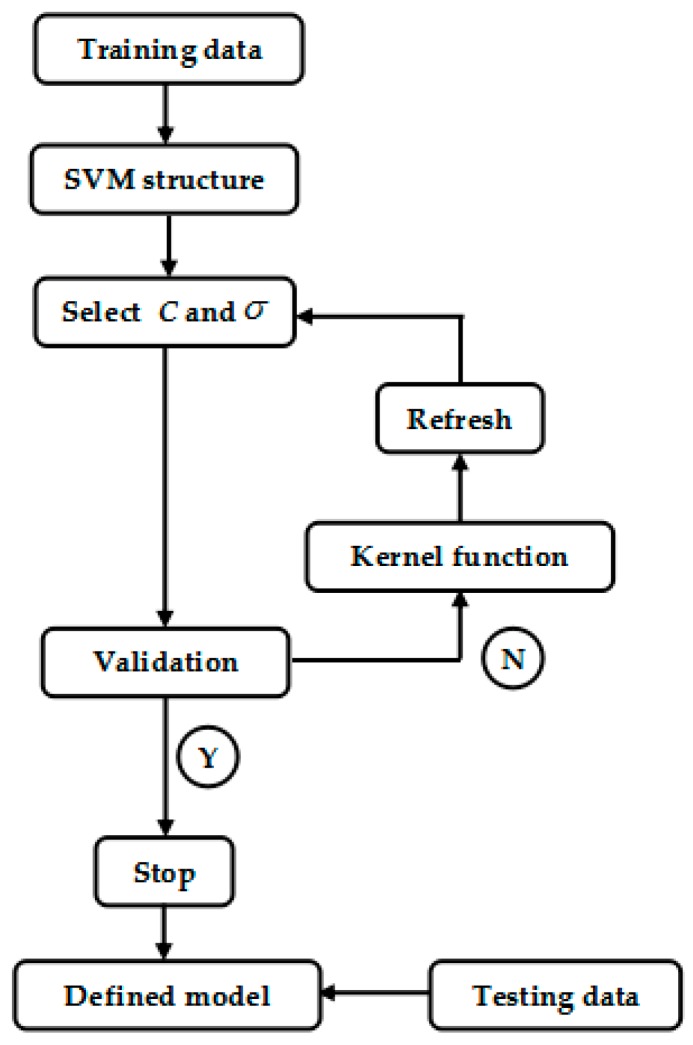
Flowchart for best parameters selection for the SVM (support vector machine) model.

Logarithmic grid search algorithm is used for both parameters. The training dataset, in which 16 cases (eight infection cases and eight non-infection cases) were used as input to the SVM to search for the best fit parameters and validated using the validation dataset. Figure 5 shows a flowchart of the search algorithm. The range of parameters C and σ are within $2^{-5},\;2^{-4},\ldots ,\;2^{5}$. For each iteration in the loop, parameters C and σ were combined. The testing dataset were applied to test the SVM model and the best testing outputs were recorded the best parameters combination.

The SVM model was trained and tested under the best parameters’ combination. To improve the generalization of the SVM model, cross-validation was applied to it. The ensemble method was not necessary since there were no initial weights and biases in the training process of the SVM model, which is similar to the ENN procedure as explained in Figure 4.

### 2.6. Evaluate the SVM Predictive Performance

The ROC curve analysis is a statistical method, which has been used in medical diagnosis since 1960. ROC curve can graphically represent the performance of a binary classification system at a particular classification threshold (discrimination threshold) that has been widely used to evaluate the performances of classifiers [46,47]. The area under the ROC curve (AUC) is the main performance index of a classification. An AUC of 1.0 implies perfect discrimination but almost nonexistent in practical. Therefore, an AUC of above 0.9 implies a pretty good classifier, and an AUC of 0.5 is equivalent to a random model, where as an AUC of less than 0.5 implies classifier not fit the actual situation and basically does not appear in practical application. Hence, the ROC curve and AUC analysis were applied to evaluate the performances of the prediction models in this study. Furthermore, the sensitivity (SEN), accuracy (ACC), and positive prediction value (PPV) were also calculated, because they are a very important index of medical diagnosing. These indices are defined by Equations (5)–(7), where TP is true positive, TN is true negative, FP is false positive, and FN is false negative.

(5) $$SEN=\;\frac{TP}{TP+FN}\;$$

(6) $$ACC=\;\frac{TP+TN}{TP+FP+TN+FN}\;$$

(7) $$PPV=\;\frac{TP}{TP+FP}$$

## 3. Results

### 3.1. Prediction Ability of the ENN Model

To estimate the generalized prediction abilities of the ENN model, testing data was applied to the model, and the outputs were used to generate the ROC curve analysis. Table 3 summarizes the ROC curves analyses for the ENN prediction models. In order to evaluate the performances of the models, the five-fold cross-validation method was applied. The results show that ENN models have high VAP recognition rates, and the average ACC was 0.9479 ± 0.0135 (mean ± SD), the average SEN was 0.9714 ± 0.0131, and the average PPV was 0.9288 ± 0.0306.

### 3.2. Prediction Ability of the SVM Model

In order to improve the identification accuracy of the SVM model, the best parameters were selected first. Testing data were applied to the model and the outputs were used to do the ROC curve analysis. Table 4 summarizes the optimal parameters for the model as C = 4 and σ = 8. The SVM model had the highest accuracy of 0.846, and the SVM model was built with best parameters combination to have a better performance for VAP detection. Table 5 summarizes the ROC curves of the analyses for the SVM prediction model. In order to evaluate the performances of the models, the fivefold cross-validation method was also applied. The results show that SVM models have good VAP recognition rates, the average ACC was 0.8686 ± 0.0422, the average SEN was 0.9250 ± 0.0423, and the average PPV was 0.8639 ± 0.0276. These data show that, the ENN model has better performance than the SVM model in recognizing VAP. 

### 3.3. ROC Curve Analysis

**Figure 6:**
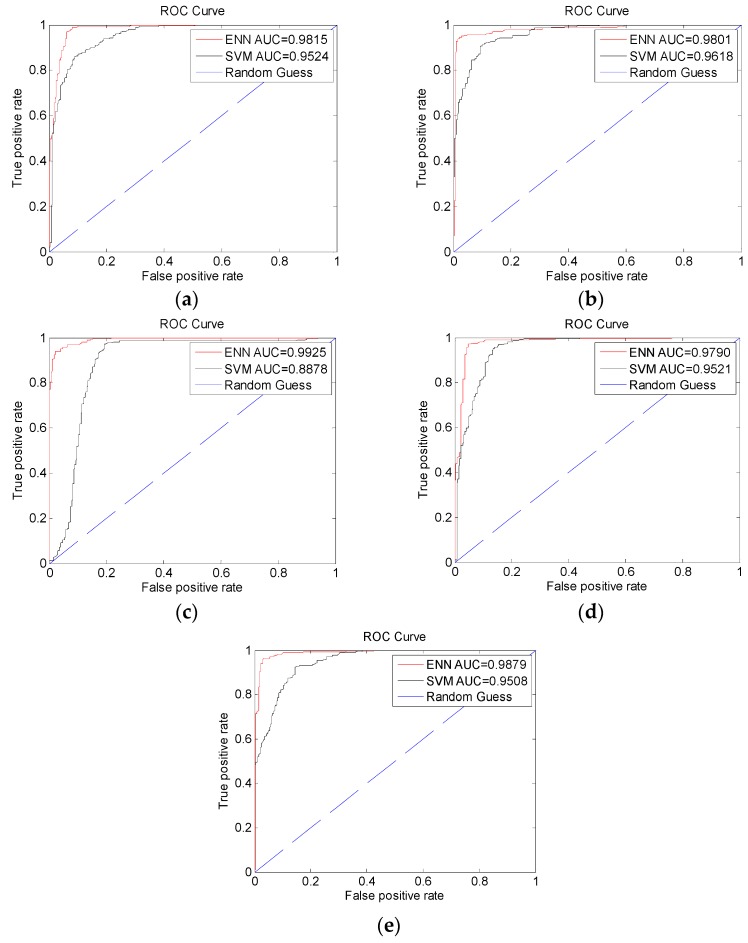
The ROC curves of ENN models: (**a**) dataset1, (**b**) dataset2, (**c**) dataset3, (**d**) dataset4, and (**e**) dataset5.

The ROC curves for ENN and SVM models were analyzed as shown in Figure 6. The average AUC of the ENN prediction model was 0.9840 ± 0.0058 (mean ± SD). The average AUC of the SVM prediction model was 0.9410 ± 0.0301. The high mean values and the low SD values confirm that the results are uniform and reliable.

## 4. Conclusions and Discussion

In this study, machine learning methods were applied to predict whether patients are infected with VAP in the ICU. ANN and SVM models were trained and tested. ROC curve analysis showed that both ANN and SVM models have high recognition for pneumonia identification. The ANN prediction model showed higher accuracy than the SVM prediction model. Both ANN and SVM methods can be used as a discriminate classifier, but SVM maps the feature distribution of training data to the feature space of higher dimensions, making the training data in the high-dimensional feature space present a linear distribution, while the output of the SVM is one or zero. In contrast, the ANN model continuously updates the weight distribution between nodes in each layer so that the actual output of training data is approximately or equal to the target output. In this way, the similarity between the actual output and the target output can be shown. When selecting different thresholds, the ANN model will produce different classification results and will produce a higher degree of recognition.

In addition, the SVM method was used to establish a model for the identification of pneumonia with *Pseudomonas aeruginosa* infection. In this study, two important parameters C and σ of the RBF were selected using grid search method, and the optimal combination of parameters was selected for training and testing the model. The test results are similar to "local optimization". In fact, the C and parameters are not limited to the range used in this study. In addition, the selection of RBF as a kernel function has produced good results. Other kernel functions are also used for SVM, such as homogeneous polynomial function, non-homogeneous polynomial function, hyperbolic tangent function and so on. In the future work, the range of SVM parameters will be expanded to cover wider search space and avoid local optimization, such as using genetic algorithm to find the best settings for the parameters C and *σ*, and different kernel functions will be tested to improve the performance of the SVM models.

Although the accuracy of the ANN model is better than SVM, the former needs higher computational cost than the latter to learn from the training data. This problem can be solved using off-line analysis with high performance computers. However, the ANN model parameters are significantly more than SVM, in particular when ensemble neural network method is used. Currently, portable devices using microprocessor are getting more and more popular. When considering memory size issue of portal devices, the SVM algorithm maybe a good solution. Hence, the two proposed algorithms can be used for the development of different VAP screening systems. The trade-off between accuracy, computational cost, and memory size is releasable with more powerful hardware coming in the future. Recently, deep learning has been applied for modeling more complex nonlinear relationships in medical researches [48,49,50] using big database and high performance computing. Therefore, through more data collection from multiple hospitals in the future, it is hoped that deep learning algorithm can be applied in early detection of multiple types of pneumonia bacteria in ICU in the near future.

*Pseudomonas aeruginosa* was used as an indicator gas of pneumonia due to limited available data. *Pseudomonas aeruginosa* was major and serious species in ICU hospital. There are mainly five kinds of bacteria including *Klebsiella pneumoniae, Acinetobacter baumanii, Escherichia coli, Staphylococcus aureus* and *Pseudomonas aeruginosa* contribute to VAP. Furthermore, according to the different pathogenic bacteria, pneumonia can be divided into viral pneumonia, bacterial pneumonia, mycoplasma pneumonia, fungal pneumonia, and other non-infectious factors caused pneumonia. In the future work, more patients’ data will be acquired, and the database will be established for further studies on VAP recognition.

The sensitivity of the sensor arrays has significant impact on the accuracy of the patients’ breathing gases detection models. The results of sensor sensitivity analysis showed that 6 out of 32 sensors have a poor performance on the identification of pneumonia and were abandoned. However, it does not mean that the identification rate for other pathogens is also poor. In the future work, sensors with less effective identification of VAP will be analyzed, and the process of sensor design will be improved. Furthermore, other pathogens will be tested to make sure that the sensor arrays have a better identification performance. In addition, traditional metal oxide sensors not only require serious operating conditions, but are easily affected by the external environment, and often cause data drift to appear or singular data occurrence. With the rapid development of semiconductor materials, nano-material sensors are booming. Nano-material with high sensitivity not only can sense individual gas molecules, but easy operating, stable, and not susceptible to external influences. Therefore, the development of grapheme and metal oxide material sensors [51,52] will be an important part of the future work.

Electronic nose with machine learning algorithms is not only lower cost, but has less requirements, is easier to operate, and responds rapidly. The process of analysis and testing can be completed in few minutes. Therefore, in this study, electronic nose was used to predict pneumonia infection, providing a rapid novel non-invasive examination and inspection method for VAP diagnosis. It is a great gospel for pneumonia patients to reduce the mortality rates. Furthermore, an electronic nose based on machine learning methods can also gradually achieve the concept of patient-centered high-quality medical care and enhance the quality of life care.

## Figures and Tables

**Table 1:** Several diseases and the corresponding indicator gases.

Common Disease	Indicator Gases
Renal disease	Ammonia, Mono-methylamine, Dimethylamine, Trimethylamine
Skin Disease	Melanoma biomarkers, Fatty acids
Diabetes	Glyceria, Acetone
Lung Cancer	Styrene, Decane, Isoprene, Benzene, Undecane, 1-hexene, Hexanal, Propyl
Asthma	Nitric Oxide (NO)

**Table 2:** Twelve patients with infection *Pseudomonas aeruginosa* and twelve patients of non-infection (WBC: White Blood Cell (10^3^/uL); PLT: Platelet (10^3^/uL); Seg: Segmented Neutrophils (%); CRP: C-Reactive Protein (mg/L); N/A: Not Available; and X: Non-infection patient).

No.	Sex	Age	WBC	PLT	Seg	CRP	Sputum	No.	Sex	Age	WBC	PLT	Seg	CRP	Sputum
1	male	87	7.38	120	61.4	2.07	*Pseudomonas aeruginosa*	1	male	87	5.27	146	58	N/A	X
2	male	90	10.23	363	70.8	N/A	*Pseudomonas aeruginosa*	2	male	83	6.18	11	86.1	N/A	X
3	female	80	13.73	258	83.1	7.09	*Pseudomonas aeruginosa*	3	male	44	20.95	194	90.4	N/A	X
4	male	63	12.05	39	91.1	21.93	*Pseudomonas aeruginosa*	4	female	51	16.25	352	89.2	3.52	X
5	male	80	31.8	286	93.5	29.12	*Pseudomonas aeruginosa*	5	female	68	9.7	191	83.9	13.05	X
6	male	54	3.25	75	93	11.43	*Pseudomonas aeruginosa*	6	male	51	6.75	178	77.3	N/A	X
7	male	59	10.62	342	75.2	5.99	*Pseudomonas aeruginosa*	7	female	53	20.56	204	93.6	0.64	X
8	male	57	9.11	154	82.8	N/A	*Pseudomonas aeruginosa*	8	male	49	17.59	309	80.7	N/A	X
9	male	49	13.23	170	82.8	N/A	*Pseudomonas aeruginosa*	9	male	49	13.15	438	80.5	N/A	X
10	male	83	12.58	142	88.7	8.61	*Pseudomonas aeruginosa*	10	male	84	25.82	274	85.5	7.86	X
11	male	79	8.98	301	73.4	N/A	*Pseudomonas aeruginosa*	11	female	70	6.86	306	40.2	N/A	X
12	female	61	10.8	174	83.6	12.47	*Pseudomonas aeruginosa*	12	female	78	23.31	206	89.6	N/A	X

**Table 3:** The ROC curve analyses by test dataset for ENN models.

Dataset	Model Type	AUC	ACC	SEN	PPV
At Best Threshold					
1	ENN Model	0.9815	0.9304	0.9929	0.8825
2	ENN Model	0.9801	0.9518	0.9571	0.9470
3	ENN Model	0.9925	0.9375	0.9679	0.9125
4	ENN Model	0.9790	0.9625	0.9714	0.9544
5	ENN Model	0.9879	0.9571	0.9679	0.9476
Average	ENN Model	0.9842 ± 0.0058	0.9479 ± 0.0135	0.9714 ± 0.0131	0.9288 ± 0.0306

**Table 4:** SVM grid search results for choosing the optimal parameters.

	σ	$2^{-5}$	$2^{-4}$	$2^{-3}$	$2^{-2}$	$2^{-1}$	$2^{0}$	$2^{1}$	$2^{2}$	$2^{3}$	$2^{4}$	$2^{5}$
C												
$2^{-5}$		0.500	0.500	0.500	0.500	0.671	0.288	0.361	0.500	0.584	0.500	0.486
$2^{-4}$		0.500	0.500	0.500	0.500	0.716	0.280	0.396	0.500	0.500	0.500	0.443
$2^{-3}$		0.500	0.500	0.500	0.500	0.254	0.282	0.500	0.500	0.500	0.661	0.500
$2^{-2}$		0.500	0.500	0.500	0.500	0.286	0.305	0.352	0.513	0.682	0.679	0.548
$2^{-1}$		0.500	0.500	0.500	0.500	0.346	0.500	0.363	0.671	0.500	0.500	0.679
$2^{0}$		0.500	0.500	0.500	0.500	0.318	0.461	0.500	0.500	0.500	0.711	0.500
$2^{1}$		0.500	0.500	0.500	0.500	0.305	0.500	0.500	0.500	0.500	0.500	0.500
$2^{2}$		0.500	0.500	0.500	0.500	0.695	0.471	0.500	0.500	**0.846**	0.500	0.707
$2^{3}$		0.500	0.500	0.500	0.500	0.695	0.500	0.500	0.575	0.500	0.500	0.730
$2^{4}$		0.500	0.500	0.500	0.500	0.695	0.500	0.500	0.546	0.755	0.500	0.786
$2^{5}$		0.500	0.500	0.500	0.500	0.695	0.500	0.500	0.500	0.743	0.500	0.8250

**Table 5:** The ROC curve analyses by test dataset for SVM models.

Dataset	Model Type	AUC	ACC	SEN	PPV
At Best Parameters					
1	SVM Model	0.9524	0.8786	0.8786	0.8786
2	SVM Model	0.9618	0.8946	0.8821	0.9048
3	SVM Model	0.8878	0.8786	0.9393	0.8376
4	SVM Model	0.9521	0.8964	0.9714	0.8447
5	SVM Model	0.9508	0.7946	0.9536	0.8536
Average	SVM Model	0.9410 ± 0.0301	0.8686 ± 0.0422	0.9250 ± 0.0423	0.8639 ± 0.0276
